# Considering Biological Sex in Traumatic Brain Injury

**DOI:** 10.3389/fneur.2021.576366

**Published:** 2021-02-10

**Authors:** Anat Biegon

**Affiliations:** Department of Radiology and Neurology, Stony Brook University School of Medicine, Stony Brook, NY, United States

**Keywords:** head trauma, concussion, men, women, sex differences

## Abstract

Published epidemiological studies of traumatic brain injury (TBI) of all severities consistently report higher incidence in men. Recent increases in the participation of women in sports and active military service as well as increasing awareness of the very large number of women who sustain but do not report TBI as a result of intimate partner violence (IPV) suggest that the number of women with TBI is significantly larger than previously believed. Women are also grossly under-represented in clinical and natural history studies of TBI, most of which include relatively small numbers of women, ignore the role of sex- and age-related gonadal hormone levels, and report conflicting results. The emerging picture from recent studies powered to detect effects of biological sex as well as age (as a surrogate of hormonal status) suggest young (i.e., premenopausal) women are more likely to die from TBI relative to men of the same age group, but this is reversed in the 6th and 7th decades of life, coinciding with postmenopausal status in women. New data from concussion studies in young male and female athletes extend this finding to mild TBI, since female athletes who sustained mild TBI are significantly more likely to report more symptoms than males. Studies including information on gonadal hormone status at the time of injury are still too scarce and small to draw reliable conclusions, so there is an urgent need to include biological sex and gonadal hormone status in the design and analysis of future studies of TBI.

## Introduction

Traumatic brain injury is a major cause of death and disability, especially among young people, and a major public health problem throughout the world. Epidemiological studies of TBI, mostly relying on emergency department and hospitalization records, consistently report higher incidence in men (1–4), sometimes explained by the higher propensity of men to be involved in physical altercations, military service and contact sports. However, this contention may need to be revised in view of recent publications suggesting that millions of women are exposed to TBI or repeated concussions caused by intimate partner violence (IPV), which are often unreported and undetected (5, 6). Similarly, in most observational and clinical studies of TBI, women represent ~30% or less of subjects (Tables 1, 2). Consequently, the natural history, outcome and pathophysiology of TBI in women in general, and IPV victims in particular, have received little systematic investigation to date. Investigation of TBI and concussion outcome in women is also complicated by the fact that during their life time, women undergo massive and abrupt changes in gonadal hormone secretion at puberty and menopause, and are exposed to fluctuating levels of the same hormone across the menstrual cycle during their reproductive years. Female and male gonadal hormones are known to exert many diverse effects on brain structure and function [reviewed in (39)] which are likely to modulate the brain response to trauma in multiple ways and may, in principle, offer sex-specific treatment targets. This is an important and timely question since with the increasing involvement of women in the military (40) and in sports, including contact sports such as Rugby (41); the number of women at risk for sustaining TBI or concussion is on the rise, while treatment algorithms are still based on the results of clinical studies with an overwhelming majority of men (70% or above), none of which resulted in an FDA approved treatment for the neurological sequelae of TBI; despite promising results from animal studies (Table 1). A comprehensive literature review of sex differences in human TBI and animal models has been published recently (42), with 73 papers demonstrating better TBI outcome in men relative to women, 41 papers which show the opposite (women better than men), 28 papers reporting no difference and 14 papers reporting mixed results. Obviously, these results are not conducive to understanding female-specific risks and attributes of TBI. The reasons for the discrepancy between human and animal studies have been addressed by us and others and are outside the scope of this minireview (42, 43).

**Table 1:** Key clinical trials in TBI.

**References**	**%women**	**Outcome**	**Analysis by Sex**
Young et al. (7)	21	3-month GOS	No
Marshall et al. (8)	24	6-month GOS	No
Marmarou et al. (9)	29	3-month GOS	No
Morris et al. (10)	23	6-month GOS	No
Clifton et al. (11)	NR	6- month GOS	NR
Maas et al. (12)	18.6	6-month GOS	No
McCarthy et al. (13)	26	3-24 month GOS	No
Giacino et al. (14)	27	4-6 week DRS	No
Zafonte et a. (15)	25	3 months GOS	No
Skolnick et al. (16)	21	6-month GOS	No
Wright et al. (17)	26	6-month GOS-E	Yes (M > F)^*^
Nichol et al. (18)	16	6-month GOS-E	No
CRASH-3 collaborators (19)	19	4-week mortality	No
Rowell et al. (20)	26	6-month GOS-E	No

*GOS, Glasgow outcome scale; DRS, disability rating scale; GOS-E, Glasgow coma scale, extended. NR, not reported. M, men; F, women. M > F better outcome in men*.

* *Trend, p = 0.07*.

*Only phase III trials including > 300 subjects are included*.

*None of these studies was analyzed for a sex x age interaction*.

**Table 2:** Representative observational studies of moderate and severe TBI biomarkers and outcomes.

**References**	**N (%F)**	**Primary outcome**	**Analysis by**	
			**Sex**	**Sex X Age**
Farin et al. (21)	957 (23)	ICP, Edema	Yes (M > F)	Yes (F < 50 worst)
King et al. (22)	159 (23)	12-month GOS	Yes (M = F)	No
Davis et al. (23)	13,247 (24)	In-Hospital mortality	Yes (M = F)	Yes (> 50 F > M)
Corrigan et al. (24)	3,444 (28)	RTW	Yes (M > F)	Yes (> 55 F > M)
Berry et al. (25)	72,294 (30.8)	Mortality	Yes (F > M)	Yes (> 45 F > M)
Ottochian et al. (26)	1,807 (22)	Mortality	Yes (M > F)	Yes (> 55 M > F)
Yeung et al. (27)	(2979) 29	Mortality, edema	Yes (M = F, mortality	NA^*^
			M > F, edema)	
Lavoie et al. (28)	175 (25)	Depression (PHQ-9)	Yes (M = F)	No
Walker et al. (29)	10,125 (27)	1-5 year GOS	Yes (M = F)	No
Puffer et al. (30)	1,169 (26)	GOS-E trajectory	No	No
Wilkins et al. (31)	304 (19)	6-24 month GOS-E	No	No
Stromberg et al. (32)	7,867 (25)	RTW	Yes (M = F)	No
Deng et al. (33)	264 (22)	ICP, edema, surgery	Yes (M = F, edema)	No
			(F > M, surgery)	
Gruen et al. (34)	164 (25)	30 day mortality	No	No
Mellett et al. (35)	429 (22)	Mortality, GOS, NRS, DRS	No	No
Okonkwo et al. (36)	1,359 (32)	GFAP, GOS-E	No	No
Xu et al. (37)	1,206 (32)	CRP, GOS-E	No	No
Kerezoudis et al. (38)	2,508 (35)	Mortality	No	NA^**^

*ICP, intracranial pressure; GOS, Glasgow outcome scale; RTW, return to work; GOS-E, Glasgow coma scale, extended; NRS, Neurobehavioral rating scale; DRS, disability rating scale, GFAP, Glial Fibrillary acidic protein; CRP, c-reactive protein*.

*NA, not applicable*,

* Study only included young women

** *All participants were elderly*.

Another emerging issue related to effects of biological sex on outcome of TBI is the recent recognition of the devastating long-term sequelae of repeated concussive TBIs (44), most notably chronic traumatic encephalopathy, a condition discovered and initially studied exclusively in male athletes (45).

Here we offer a concise critical review of emerging data on the effect of biological sex and hormonal status on TBI incidence and outcome, highlighting some possible mechanisms and identifying significant knowledge gaps which need to be filled in order to improve outcome of TBI.

## TBI Incidence and Prevalence in Men and Women

Traumatic brain injury is a major public health concern and prominent cause of death and disability. Worldwide, in 2016, there were ~27 million new cases of TBI with an age-adjusted incidence rate of 369 per 100,000—representing a 3.6% increase from 1990. In the same year, prevalence was 55.5 million individuals, representing an 8.4% increase from 1990 (46). In the US, TBI statistics published by the Centers for Disease Control and Prevention (1–3, 47) show that the combined rates for TBI-related emergency department (ED) visits, hospitalizations, and deaths in the United States have been on the rise and totaled 823.7 per 100,000 US population in 2010. Furthermore, an estimated cumulative 5.3 million individuals are living with a TBI-related disability in the United States. This represents a prevalence of ~2% of the U.S. population (47).

Epidemiological studies consistently report higher incidence in men, such that the odds of sustaining a TBI are 2.22 times higher in men than in women (4). The reported TBI prevalence in the general population is 16.7% among males and 8.5% among females. Overall, males account for ~59% of all reported TBI-related medical visits in the United States (48). This robust and consistent sex difference is sometimes explained by the higher propensity of men to be involved in physical altercations, military service, and contact sports (4). Sex differences in TBI incidence are modulated by age, and recent reports show that among the elderly (over 65), overall TBI incidence (49) and rates of ED visits for mild TBI were higher for women than for men (50). Similarly, rates of sports related injuries in young women seem to be equal or higher to those of men [e.g., (51–54), Table 3].

**Table 3:** Representative studies of mild TBI and sports concussion incidence and outcomes.

**References**	**N (%F)**	**Primary outcome**	**Analysis by**	
			**Sex**	**Sex and Age**
Colvin et al. (55)	234 (60.3)	PCS, IMPACT	Yes (M > F)	NA
Preiss et al. (56)	260 (34)	PCS	Yes (M > F)	Yes (MinorF > adultF)
Bazarian et al. (57)	1425 (45.1)	RPQ, PCS	Yes (M > F)	Yes (F < 55 worst)
Covassin et al. (58)	296 (31.4)	IMPACT, PCS	Yes (M > F)	NA
Styrke et al. (59)	163 (31.8)	RPQ 3 years	Yes (M > F)	No
McMahon et al. (60)	375 (29.9)	GOS-E, BSI-18, RPQ	No	No
BluMfeld et al. (61)	1500 (40)	Incidence, Symptoms, duration	Yes (M > F)	No
Ma et al. (62)	108 (100)	Incidence	NA	NA
Albanese et al. (63)	53 (50.8)	PCS, ASI-3, DTS, NSI	Yes (M > F)	No
Brickell et al. (64)	172 (50)	NSI, PCL-C	Yes (M > F)	NA
Chandran et al. (65)	580 (51)	Incidence, duration	Yes (M > F)	NA
Harrold et al. (66)	426 (58)	SCAT3 and K-D	Yes (M > F)	No
MacDonald et al. (67)	94 (7)	5 year GOS-E	No	No
Mollayeva et al. (68)	94 (38)	Pain	Yes (M = F)	No
Roos et al. (51)	3825 (59)	Incidence	Yes (M > F)	No
Rosene et al. (52)	415 (30)	Incidence	Yes (M = F)	No
Bahraini et al. (69)	4012 (5.4)	NSI	Yes (M > F)	NA
Lippa et al. (70)	158 (50)	NSI, PTSD, AIS.	Yes (M > F)	NA
Terry et al. (71)	1265 (42)	Symptom duration	Yes (M > F)	No
Varriano et al. (72)	436 (42.6)	PCS rate	Yes (M > F)	Yes (Young > old)
Nelson et al. (73)	1154 (34.4)	GOS-E 3, BSI-18, RPQ	No	No
Yue et al. (74)	100 (29)	GOS-E	Yes (M > F)	NA
Combs et al. (75)	494 (44.7)	Graded symptom checklist	Yes (M > F)	NA
Kennedy et al. (76)	184 (10.3)	Depression	Yes (M > F)	NA
Putukian et al. (77)	1922 (33.8)	Incidence, duration	Yes (M > F)	NA
Spano et al. (78)	778 (11.4)	Incidence, Symptoms, duration	Yes (M > F)	No

*PCS, post-concussion syndrome; IMPACT, Immediate Post-concussion Assessment and Cognitive Testing; RPQ, Rivermeade Post-concussion Questionnaire; BSI-18, Brief Symptom Inventory 18; AsI-3, Anxiety Sensitivity Index-3; DTS, Distress tolerance scale; NSI, Neurobehavioral Symptom Index; PCL-C, PTSD checklist, civilian version; SCAT-3, Sport Concussion Assessment Tool 3; K-D, King-Devick scale; GSC, Graded symptom checklist*.

*NA, not applicable, all participants were young*.

Importantly, studies relying on reported injuries and ED visits likely paint a distorted picture of the actual incidence of TBI, since they do not include TBIs suffered by female victims of intimate partner violence (IPV). IPV is a highly gendered behavior, such that the majority of perpetrators are men and the majority of victims are women, and TBIs suffered in this context are often unreported (79, 80). The Centers for Disease Control and Prevention (81) report that 32 million women in the United States have experienced IPV during their lifetime. Moreover, the National Intimate Partner and Sexual Violence Survey states that nearly one in four women in the United States have experienced severe physical violence (being hit, kicked, choked, beaten, burned, stabbed, or shot) during their lifetime by an intimate partner (82). Many of these violent attacks are likely to result in traumatic or anoxic brain injury, since it is common for abusers to target the victim's face, neck, and head (83, 84), with the prevalence of IPV-related TBI estimated as 60 to 90% (79, 85). A recent study found that more than 80% of IPV victims referred from homeless or domestic violence shelters sustained multiple TBIs, 84% had clinically significant symptoms, yet only 21% sought medical attention at the time of injury (85). This very low rate reflects the fact that many battered women may never go to the Emergency Department or get treated by health care providers (86–89) resulting in underreporting and poor detection. Given the numbers of women over the age of 15 experiencing IPV, there could be more than 31,000,000 women who have received a traumatic brain injury (90) in the US today- a number which should radically change our perception of TBI demographics.

## Biological Sex and TBI Outcome

TBI outcome is highly variable: Moderate and severe injuries may result in death, persistent vegetative state, severe disability, moderate disability, or good recovery, which form the basis for the most commonly used TBI outcome scales—initially the 5 step Glasgow outcome scale, (GOS), more recently replaced by the 8 step extended GOS (GOS-E). Mild TBI and concussion, which actually account for the majority (~75%) of TBI cases (2), rarely result in death or severe disability but are often associated with long term changes in cognition and behavior (91, 92), which can be assessed by scales such as the Rivermeade post-concussion questionnaire [RPQ, (22, 93)]. Early studies which established risk factors for poor outcome in TBI, such as advancing age (94) did not report analyses by sex.

The influence of biological sex on the outcome of TBI has been the subject of several analyses with contradictory results, possibly due to the relatively small number of women and girls in clinical studies, lack of information on hormonal status and the wide disparity in outcome measures used for the comparison, which included such disparate measures as return to work, bacteremia and mortality [(42); Tables 1, 2]. Thus, studies comparing men and women without paying attention to hormonal status or age report no differences in outcome, better outcome in women or better outcome in men (27, 95–99). Probably for the same reason, the recognition of various risk factors for poor TBI outcome, including advanced age, is biased toward men, and this has not changes since age as a risk factor for poor TBI outcome was first reported by Teasdale et al. (94). Consequently the treatment guidelines for head injury [e.g., (100, 101)] are heavily influenced by findings in male patients. Differences between the sexes in the frequencies of risk factors and their effect on early and late outcome as measured by TBI-specific outcome scales (i.e., GOS and GOSE) have not been systematically investigated to date. Similarly, research on mild TBI and concussion, which actually account for the majority (~75%) of TBI cases (2) in the general population is also plagued by small studies, disparate outcome measures and paucity of women (see Table 3). Understandably, mild TBI/concussion is more highly prevalent among athletes and the military (102, 103). While studies focusing on contact sports in which women are not represented (91, 104, 105) do not contribute to the question at hand, there is a recent explosion of publications on sex differences in incidence and outcome of sports related concussions in sports in which women do engage (Table 3). These studies include a higher proportion of women relative to clinical and observational studies moderate—severe TBI (Tables 1, 2) and despite the wide range of sports included, from water polo through rugby to Jio-Jistsu, and the very large variation in outcome measures (Table 3), there appears to be a near consensus that women are more likely to receive concussions in sports and have a worse outcome. However, these studies cannot be generalized to the population at large since they typically include only young healthy subjects engaging in sports (Table 3).

### Biological Sex, Age and TBI-Related Mortality

The picture is somewhat less confusing if we focus our attention on relatively large (N ≥ 1000 total) studies reporting “hard,” completely objective outcome measures such as mortality and persistent vegetative state which segregate outcome by both age and sex. An early example is the community study published by Klauber et al. (95), which reported no effect of sex on mortality. However, upon perusal of the breakdown of mortality data by sex as well as age (in decades), The results show that between puberty and old age, there is no significant effect of age on mortality in women, while mortality in men shows a strong and highly significant association with increasing age, as would be expected from prior studies (94). This sex x age interaction on the outcome of TBI results in a reversal of a sex difference in TBI mortality, which occurs around age 50, thereby negating an overall effect of sex on outcome. Thus, between the ages of fifteen and fifty, men have a (small) mortality advantage over women in the same age groups, but this is reversed after age fifty, when men are significantly more likely to die relative to younger men or women in all age groups. This pattern emerged in the absence of sex differences in injury severity (95). Assuming age is a reasonable surrogate for hormonal status in the absence of actual data, age 15 and over may be considered to be post pubertal and women over 50 may be considered to be mostly post-menopausal. This age cutoff is commonly used to separate mostly pre- and mostly postmenopausal female patients because the majority of women reach menopause during the decade between 45 and 55 years of age (106, 107), whereas in men a continuous decline in testosterone levels is associated with ages >50 years (108).

In a subsequent study involving 25,300 emergency head-related admissions, it was found that women were more likely to die from all head injuries (OR = 1.3) with an even higher likelihood of death from violent head injuries (OR = 2.38). The authors also note that women 15 or older stayed in the hospital longer than men (109).

Davis et al. (23) published a study of a total of 13,437 patients (*n* = 3,178 females and 10,259 males) with moderate-to-severe TBI (head AIS > or = 3) from a county trauma registry. While overall mortality was similar in men and women, a separate analysis performed for premenopausal (<50 years) vs. postmenopausal (> or = 50 years) patients, after stratification by decade of life, revealed no statistically significant difference in mortality of pre-menopausal females vs. males, though outcome was significantly better in postmenopausal females vs. males (OR 0.63, 95% CI) with similar rates of hypotension (Systolic blood pressure <90 mm Hg), head Abbreviated Injury Score (AIS), and Injury Severity Score (ISS). Stratification by decade of life revealed the gender survival differential inflection point to occur between ages 40-49 (OR 1.06, 95% CI 0.66-1.71, *p* = 0.798) and ages 50-59 (OR 0.38, 95% CI 0.20-0.74, *p* = 0.005). The authors then conclude that endogenous female sex hormone production is not neuroprotective in human TBI. These results also dovetail with those of studies performed more recently (25, 27). Berry et al. (25) examined records of 72,294 moderate and severe injury patients from the National Trauma Database (2000–2005) and found that peri- and postmenopausal women (Age more than 45) demonstrated improved survival relative to men, but premenopausal women did not. The exception to this trend is the (relatively modestly sized) study by Ottochian et al. (26); which included 1,800 subjects (22% women) and reported a survival advantage for men over 55 relative to women over 55. The study by Yeung et al. (27), which included women under 45, reported no survival advantage in “hormonally active” women as compared to men in the same age range. Consequently, authors of both papers concur that estrogen does not appear to confer neuroprotection in women after TBI.

### Sex Differences in Mild TBI/Concussion Outcome

Sex differences in concussion incidence and outcome were reviewed by Dick (110) who concluded that the literature supports higher incidence and worse outcome in women. A later review (111) opined the literature reviewed did not support this conclusion. Table 3 features representative subsequent studies, a few of which stratified data by sex as well as age. Thus, in a 2010 study, Bazarian et al. (57) examined mTBI outcome in 1425 mTBI patients (45.1% female) presenting to an academic emergency department. Men were significantly less likely to be in a higher Post concussive symptoms (PCS) score category relative to women (OR = 0.62), and this association was more prominent during child-bearing years (between puberty and menopause) for females. The authors conclude that female sex is associated with significantly higher odds of poor outcome after mTBI, as measured by PCS score, after control for appropriate confounders. This conclusion resonates with the results of a 2009 study (56) reporting no sex difference in post-concussion symptoms among minors (presumably mostly pre-pubertal subjects) with worse outcome in adult women (Table 3). This study did not include women older than 50. This is common in many of the more recently published studies on concussion in men and women performed in athletes engaged in a variety of sports and in military personnel, and therefore including a relatively young population (Table 3). With this caveat in mind, there appears to be a near-consensus that across different sports, women are more likely than men to suffer sports-related concussions, report more symptoms, have a slower recovery and overall more negative outcome. The latter observations were also reported in an exquisitely designed study focusing on female service members (64), whereby women (*N* = 86) reported more symptoms despite having been matched with the male comparison group (*N* = 86) for TBI severity, mechanism of injury, bodily injury severity, days post-injury, age, number of deployments, theater where wounded, branch of service, and rank. A pilot publication on 100 subjects with mTBI (29% women) from the TRACK TBI study examined PTSD as an outcome and concluded that sex may interact with age for PTSD symptomatology, with females 30–39 y at highest risk (74). The authors conclude that prevention and rehabilitation/counseling strategies after mTBI should likely be tailored for age and sex. Rather disappointingly, larger studies published using TRACK-TBI data, while including a similar percentage of women, did not use sex as a grouping variable in the analysis of outcomes [(60, 73), Table 3].

### Neurodegenerative Disease Following Single or Repeated TBI in Men and Women

Traumatic brain injury is believed to be an important risk factor for neurodegenerative diseases, such as Alzheimer's disease (AD) and chronic traumatic encephalopathy (CTE) (112, 113). Despite the fact that AD incidence and prevalence is significantly higher in women relative to men, and significant sex differences in the disease trajectory and response to treatment (114–116), there is no information on whether the AD risk associated with TBI is modulated by biological sex.

Chronic traumatic encephalopathy (CTE), a dementia-like syndrome which manifests at younger ages than AD, appears to be linked to repeated exposure to Mild TBIs/concussions rather than a single TBI and is associated with an anatomically distinct pattern of tau deposition in the absence of significant amyloid deposits (105, 117). This condition was initially characterized and subsequently studied exclusively in male athletes and military personnel (44, 45, 118–120). Consequently, there are no reports on TBI-related CTE in women since the modern definition of this entity. Tantalizingly, the only description of CTE-like brain pathology in a woman is a case study published by Roberts et al. (121) titled “Dementia in a punch-drunk wife,” describing a woman who died following prolonged and severe IPV.

## Possible Mechanisms Underlying Sex Differences in TBI Outcome

The mechanisms underlying the relatively poor outcome of young women with TBI and concussion are not known, though several suggestions have been made based on small studies in mild TBI. Thus, Albanese et al. (63) propose that higher anxiety sensitivity mediates gender differences in post concussive symptoms, and another study cites higher preinjury migraine rates in women as a reason for longer time to return to school and sports among concussed female athletes (71). Attempts to use a similar approach regarding depression yielded conflicting results (28, 76). Yue et al. (74), summarizing results from the TRACK-TBI pilot, make the general observation that the sex differences they observe “may be attributable to cortical maturation, biological response, social modifiers, and/or differential self-report” although suspected sex differences in the latter variable have not been consistent when examined in the athlete population (122, 123). In addition, Alsalaheen et al. (124) and Grafton et al. (125) invokes different strategies to stabilize the head in response to impulsive loads as a possible explanation for sex differences in concussion injury risk. Recent studies also suggest sex differences in biomechanics of concussion in sports (126). However, these studies usually report higher impacts in males and it is hard to see how these findings can explain the consistent findings of worse outcome in women.

### Effects of Gonadal Steroid Levels

Both female and male gonadal hormones are known to exert multiple diverse effects on brain structure and function; which can be roughly divided into irreversible (organizational) effects during brain development and reversible (activational) effects after puberty [reviewed in (39)]. Numerous, though not all, animal studies suggested that female sex hormones improve brain injury outcome [Reviewed in (42, 127)]. These animal studies led to a series of clinical trials of progesterone in human TBI, however the pivotal phase III trials failed to provide any evidence of improvement in outcome [(16), Table 1]. This was the only human study in which gonadal steroid levels were manipulated through hormone administration in men and women. Interestingly, the studies were not designed to examine outcome by sex. In a study of the relationship between endogenous progesterone levels and menstrual cycle phase in women (128), the authors found that women injured during the luteal phase of their menstrual cycle, when progesterone concentration is high, had significantly lower General Health Ratings and higher RPQ somatic scores one month after injury than women injured during the follicular phase of their cycle, suggesting that high ambient levels of female gonadal steroids have a negative rather than a positive effect on mTBI outcome. In a similar vein, estradiol was identified as a “potent mortality marker,” with strong relationships between increased serum E2 levels and elevated mortality risk after severe TBI reported by Wagner et al. (129). NB, the study populations was mostly male and results were not analyzed be sex.

### Sex Differences in Brain Volume

Total intracranial volume has been shown to be an independent predictor of the effect of TBI on intelligence, in accordance with the cognitive reserve theory (130). In a similar vein, Ystad et al. (131) reported a highly significant correlation between hippocampal volume and performance of a verbal memory (CVLT) task, and Umile et al. (132) confirmed the vulnerability of the medial temporal lobe to mild TBI, which correlated with neuropsychological deficits. A recent study of cognitive outcome in TBI demonstrated a significant declines (relative to individual premorbid intelligence) in abstract reasoning as measured by Raven's progressive matrices–R (RPM-R) in moderate-severe as well as mild TBI (133). In this study, there was a highly significant correlation between the volume of the insula and deficits in RPM-R performance. These studies did not report data from women.

Sex differences in intracranial volume, brain size and regional size, are found from birth and are thought to reflect organizational effects of gonadal steroids which occur during fetal brain development. On average, men have a larger brain size than women as denoted by a higher intracranial volume (ICV) and total brain volume (~8–15% larger volumes in men), higher tissue/region-specific volume (134), a greater amount of neurons, increased global cortical thickness and larger total cortical surface area relative to women (135–137). In a more recent article which compared 58 young women and 44 young men, Martinez et al. showed ICV and total brain volume were highly significantly smaller in women relative to men (*t* = 8.22, *p* < 0.00005 and *t* = 7.61, *p* < 0.00005, respectively). Importantly, the size of the sex difference in regional brain volumes diminishes with advancing age (138).

A similar sex X age interaction was observed in several regional studies: In an early study focusing on the corpus callosum (139), the cross sectional area of the corpus callosum and splenium was measured off a midsagittal MRI image from subjects with Alzhemier's disease (AD), age matched elderly controls, and young controls. Analysis of the healthy control data by sex and age shows reduction in callosal area with age in men which is not observed in women, resulting in a reversal of the sex difference seen in young controls (men > women) when the comparison is performed in elderly subjects (Women > men). In another study focusing on hippocampal volume, analysis of the healthy control population in the ADNI data base (115) showed that hippocampal volume (mean(STD) 7175 (886) mm^3^ *N* = 187 women vs. 7539 (935) mm^3^, *N* = 192 men) is slightly (5%) but significantly (*p* < 0.0001) higher in elderly men relative to women, while the sex difference in intracranial volume was 12.7% [mean 1423 cc in women and 1604 in men, (115)]. These results dovetail with long-standing evidence of earlier and steeper age-related declines in brain regional volumes in men relative to women (140–142) which has also been confirmed for the hippocampus. For example, in a study of hippocampal volume in early adulthood [39 men and 41 women, age 18–42 years, (143)], a significant negative correlation with age for both left and right hippocampus was found in men (*r* = −0.47 and−0.44, respectively) but not in women (*r* = 0.01 and 0.02, respectively). The volume decline in men appeared to be linear, starting at the beginning of the third life decade and ~1.5% per annum.

Investigations of moderate and severe TBI have demonstrated significant brain atrophy over the first year after injury in many brain regions, even those that are remote from direct injury, including the cingulate gyrus and the hippocampus (144–148). As in most other studies of TBI, the number of women included in these rather small studies did not support analysis of a sex x age interaction. To elaborate, the Schonberger (147) study included 74 men and 24 women, mostly under 50, and the Zhou study (148) examined 27 men and only 5 women.

Taken together, the sex x age interaction on TBI outcome and brain volumes described above supports the notion that women, possibly due to their smaller total and regional brain size (smaller brain reserve), have a worse outcome of moderate-severe as well as mild TBI/concussion compared to men; but this difference may be diminished or even reversed with advancing age since brain reserve is diminished at a steeper rate in men relative to women.

### Sex and Age Differences in Brain Swelling

The well-documented effects of female gonadal hormones on fluid balance (149–151) and the high frequency of idiopathic intracranial hypertension in premenopausal female patients (152, 153) supports the likelihood of differences between the sexes in frequencies of brain swelling and intracranial hypertension following TBI, specifically in presumably premenopausal women (< 50 years of age). Brain swelling (edema) and the resultant increase in intracranial pressure are known risk factors for poor outcome in humans as well as in animal models of TBI (154–159). If TBI-related swelling and ICP time-dose are indeed influenced by sex and hormonal status, i.e., higher in young women than in post-menopausal women, this could be another contribution to the sex x age interaction on outcome described above.

The Tirilizad study was one of the first clinical studies in TBI to include outcome, CT and intracranial pressure data indicative of brain swelling in a study population large and diverse enough to enable statistically powered comparisons of brain swelling between young and >50 male and female patients with moderate/severe head injury (8, 21). Overall, female patients had a significantly greater frequency of brain swelling visualized on CT than male patients−35% compared with 24% (*p* = 0.0008). This increased frequency was characteristic of premenopausal women (<51 years of age), who had a 38% rate of swelling compared with 24% among their male counterparts (*p* = 0.002), which did not change with age (21). The frequency in postmenopausal female patients (> 50 years of age) was comparable to the frequency in men. Subsequent analysis showed that the increased frequency of brain swelling in female patients was not due to higher injury severity or other confounders including advanced age, presence of subarachnoid hemorrhage (SAH), or systemic hypotension. Further analysis of the relationship between intracranial hypertension (defined as an ICP >20 mm Hg during >25% of the time it was monitored) and sex demonstrated a significantly greater frequency of intracranial hypertension among female compared with male patients (39% compared with 31%; *p* < 0.03). The sex-related difference in frequency was even more dramatic in the population <50 years (40% compared with 30%; *p* < 0.02). The increased frequency of intracranial hypertension in women and girls was not due to increased injury severity. As was the case with brain edema, the difference in rates of intracranial hypertension between the sexes was most significant among the less severely injured patients (GCS scores of 7 or 8 [33% compared with 20%; *p* < 0.02]) (21).

The findings from the Tirilizad study were corroborated in a more recent international study of TBI, showing that brain edema was associated with female sex (*P* = 0.02), and the odds of brain edema in females were greater than for males in a cohort of young subjects recruited in Hong Kong (27). The second cohort included in this study, recruited in Australia, demonstrated a smaller sex difference in the same direction which did not reach statistical significance. This study recruited subjects in the age range 12–45 so that only premenopausal females were included.

## Summary and Conclusions

Research conducted in the last couple of decades has significantly improved our understanding of the impact of biological sex on TBI incidence and outcome. However, some glaring still exist due to the slow and incomplete acceptance of the imperative to include women in TBI studies and report results stratified by sex, which need to be proactively addressed in the future.

### Key Findings

- There is increasing recognition of the high prevalence of TBI among the tens of millions of women who live with domestic violence and fail to report- or seek medical attention for- their injuries.
- Recent studies of TBI outcome which include adequate numbers of women challenge the long held view (based on animal studies) that reproductive-age women, by virtue of high levels of estrogen and progesterone, are likely to have a better TBI outcome relative to men.
- Accumulating evidence shows that reproductively competent women (after puberty and before menopause) are at higher risk for poor outcome, while postmenopausal women fare better than men of similar age (>50 years old), whose outcome worsen with age.

### Knowledge Gaps

- While recent findings suggest an important contribution of gonadal hormone levels to clinical outcome of TBI of all severities, these variables are not assessed or measured in the overwhelming majority of TBI studies.
- The safety and efficacy of old and new TBI interventions in women across the life span is unknown
- The importance of risk factors for poor outcome of TBI, established in mostly-male populations, is largely unknown in women

### Next Steps

- Female subjects with TBI need to be proactively sought out and recruited from domestic violence shelters and agencies.
- Results of clinical and research studies on TBI need to be stratified by sex and gonadal hormone status.
- Female TBI victims need to be queried about their hormonal status, i.e., Pre- or post-pubertal, pre- or post-menopausal and if reproductively competent, estimated stage of menstrual cycle (last menstrual period).
- Both men and women can benefit from actual acute and repeated measurement of sex steroid levels, including androgens, estrogens, progesterone, in order to understand possible sex-specific impact of TBI on reproductive health and possibly provide new sex-sensitive guidelines and sex-specific hormone-based treatment targets.

## Author Contributions

AB conceived of and wrote the paper.

## Conflict of Interest

The author declares that the research was conducted in the absence of any commercial or financial relationships that could be construed as a potential conflict of interest. The handling editor is currently organizing a Research Topic with the author AB.
